# Relationship between interviewer-assisted international prostate symptom score and other objective measures of bladder outlet obstruction in Southeast Nigeria: a cross-sectional study

**DOI:** 10.11604/pamj.2023.46.87.37303

**Published:** 2023-11-21

**Authors:** Solomon Kenechukwu Anyimba, Ikenna Ifeanyi Nnabugwu, Louis Tochukwu Okolie, Francis Oyiogu Ozoemena

**Affiliations:** 1 Urology Unit, Department of Surgery, University of Nigeria Teaching Hospital Ituku-Ozalla, PMB 01229, Enugu, Nigeria,; 2 Department of Surgery, College of Medicine, University of Nigeria Enugu Campus, Enugu, Nigeria

**Keywords:** Lower urinary tract symptoms, international prostate symptom score, benign prostatic hyperplasia, prostate volume

## Abstract

**Introduction:**

there are concerns that interviewer-assisted administration of the International Prostate Symptom Score (IPSS) may introduce bias to the extent that values obtained may not correlate with the more objective measures of bladder outlet obstruction (BOO) in benign prostate enlargement (BPE). This study aims to determine the relationship between interviewer-assisted IPSS and the more objective peak urine flow rate (Qmax) and postvoid residual urine volume (PVR) in men with lower urinary tract symptoms (LUTS) due to BPE in a low-resource setting.

**Methods:**

a cross-sectional study from July 2020 to June 2021. Using systematic random sampling, men ≥ 40 years old with LUTS attributable to uncomplicated BPE were recruited. Participants completed the English-language IPSS questionnaire with the needed assistance from the interviewer. Thereafter, the Qmax was assessed using uroflowmetry while PV and PVR were assessed using ultrasonography. Age, serum total prostate-specific antigen (tPSA), body mass index (BMI), and the highest level of formal education attained were determined. Multivariate logistic regression analysis was used to examine the relationship between these variables and IPSS.

**Results:**

in all, 170 men of mean age 63.7±9.9 years participated. The mean PV, PVR, and Qmax were 70.84±39.50 cm^3^, 77.66±69.30 cm^3^, and 20.25±9.70ml/s, respectively. Of these 170 participants, 134 (78.8%) attained formal education beyond the primary level. Increasing points of interviewer-assisted IPSS have a strong relationship with worsening self-perceived quality of life due to LUTS (r: 0.76; p= 0.001), but a rather weak relationship with decreasing Qmax (r: -0.40; p= 0.009) and increasing PVR (r: 0.49; p= 0.005). Higher formal education was associated with lower IPSS at presentation and was statistically significant (p = 0.004). There were no predictable relationships between IPSS and age, tPSA, PV, and BMI (p > 0.05).

**Conclusion:**

interviewer-assisted IPSS can be relied upon, but with some caution, in low-resource, low-formal education settings to give clinical information consistent with the objective measures of BOO.

## Introduction

Benign prostatic enlargement (BPE) is considered one of the most important health issues in men as they advance in age and it usually manifests as an obstruction to urine outflow known as bladder outlet obstruction (BOO) [1]. Men who develop BOO due to BPE usually experience lower urinary tract symptoms (LUTS) of the storage and voiding categories which are objectively measured using the internationally validated International Prostate Symptom Score (IPSS) and urine flow studies [1]. A diminishing stream of urine and increasing postvoid residual urine volume (PVR) suggest an increasing degree of BOO with resultant worsening LUTS [2]. While peak urine flow rate (Qmax), is an objective parameter assessed through uroflowmetry as an index of the urine stream, PVR is an index of the effectiveness of bladder emptying assessed through transabdominal ultrasonography. These parameters are required to corroborate the IPSS [2]. Some studies suggest a correlation between IPSS and these objective measures of BOO namely Qmax and PVR [3,4], but others suggest that whatever correlation exists is at best weak [5].

Though many men in low-resource settings tend to endure LUTS from an enlarged prostate to an end event such as acute or chronic urinary retention, a good proportion does present earlier with LUTS only [6]. For these men who present earlier, it is recommended that treatment decisions be guided by the reported IPSS, the perceived quality of life due to the LUTS, findings from uroflowmetry especially Qmax, and the PVR [7]. When considered collectively, these subjective and objective measures of the degree of BOO in uncomplicated BPE help in shared decision-making, ensuring that appropriate individualized therapeutic strategies are instituted [7]. To what extent do these factors agree with each other in low-resource and low-formal education settings where some assistance is required with understanding the items in the IPSS questionnaire?

The aim of this study, therefore, is to determine the nature of the relationship between interviewer-assisted administration of IPSS for assessing LUTS, the perceived quality of life due to LUTS, the peak urine flow rate (Qmax), and the PVR in a group of men in a low-resource, low-formal education setting presenting with LUTS from uncomplicated BPE. The findings from this study will speak to the rational deployment of these parameters in settings that may not have the capacity to generate all parameters for every one of such men, and that may not rely confidently on visual prostate symptom score (VPSS) as an alternative to self-administered IPSS [8,9].

## Methods

**Study design and setting:** a cross-sectional analytical study was conducted between July 2020 and June 2021 at the University of Nigeria Teaching Hospital, Enugu, a tertiary urology care center in southeast Nigeria. It was estimated that a total of 624 to 750 new cases of men with LUTS from BPE were attended to per annum in the tertiary health institution. This estimate was based on records of approximately 12 to 15 new cases of LUTS due to BPE weekly.

**Inclusion criteria:** all male patients who were aged 40 years and older who presented for medical care with LUTS, and were newly found to have BPE.

**Exclusion criteria:** all patients with mild LUTS and IPSS of less than 8 points were excluded. In addition, patients who have a neurologic deficit that could affect bladder function, have other pathologies that could explain LUTS such as bladder calculus, urethral stricture, or have serum total prostate-specific antigen (tPSA) greater than 4ng/ml were not included in the study. Also excluded were patients who had any of the recognized complications of BPE, or BOO, and those on α1 blocker or 5α reductase inhibitor, or a history of transurethral resection of the prostate (TURP).

**Variables:** the variables of interest were IPSS as obtained through interviewer assistance, the patient´s self-perceived quality of life due to LUTS score, prostate volume (PV), PVR, peak flow rate (Qmax), and tPSA. In addition, the age, body mass index (BMI), and the highest level of formal education of participants were ascertained.

**Data sources/measurement:** the level of formal education attained was taken as the point at which the participant exited formal education in the “primary (6 years), secondary (6 years), tertiary (≥4 years)" system of formal education [10]. Participants completed with necessary assistance from pre-trained researchers who were urology trainees of more than 5 years, the validated IPSS questionnaire. The participants also responded to the self-perceived quality-of-life (QoL) due to the LUTS question that forms part of the IPSS questionnaire [11]. The QoL due to the LUTS question assesses the participant´s impression of the worth of his life considering the LUTS being experienced. The total IPSS score was classified, according to the classification of the AUA, into three categories: mild (1-7 points) (which was excluded), moderate (8-19 points), and severe (20-35 points). Thereafter, transrectal ultrasonography assessment of the PV and its other echo features, and transabdominal ultrasonography assessment of PVR and median lobe protrusion were determined. Postvoid residual volume (PVR) was defined as the volume of urine remaining in the bladder after the completion of micturition. In addition, a uroflowmetry assessment of peak urine flow rate (Qmax) was done. For purposes of analyses, Qmax ≤15ml/s defined bladder outlet obstruction [12]. The PVR and median lobe protrusion were assessed using a 3.5MHz curvilinear ultrasound probe on a GE Versana Essential® (General Electric Company USA) ultrasound machine, while the Qmax was assessed using EPSON LX-310® (ARK Meditech System, India) uroflowmeter.

**Sample size:** using an appropriate formula [13], an expected correlation coefficient of 0.225 [12], and adopting a 95% confidence interval, a power of 80%, and a data error rate of 10%, the calculated sample size was 167 participants. That came to 14 participants per month. Adopting a systematic sampling technique, 1 in every 3 eligible candidates was recruited after obtaining written informed consent. From July 2020 to June 2021, a total of 170 participants were recruited.

**Statistical analyses:** hypothesis testing with the Kolmogorov-Smirnov test revealed that age was normally distributed whereas IPSS, self-perceived QoL, PVR, and Qmax were not. Mean and median were determined for these variables of interest. Spearman´s correlation was used to study the association between PVR and IPSS, and between Qmax and IPSS. Continuing, the PVR and IPSS of participants with Qmax >15ml/s were compared respectively to the PVR and IPSS of those with Qmax ≤15ml/s using a comparison of means. Multivariate logistic regression analysis was done to determine which factors among age, formal education attainment, BMI, PV, PVR, Qmax, median lobe protrusion, and tPSA independently predict IPSS as determined via interviewer assistance at presentation. The dependent variable IPSS was transformed into a normal variable for the regression analysis. Significance was set at p < 0.05. All analyses were done using IBM SPSS (IBM Co., Armonk, NY, USA) version 23.

**Ethical considerations:** the University of Nigeria's Bioethics Committee approved this study. Ethical clearance certificate number NHREC/05/01/2008B-FWA00002458-1RB00002323. All participants gave written informed consent, and the study was conducted in accordance with the Helsinki Declarations and reported in conformance with STROBE.

## Results

In all, 170 participants completed the study. Of these, 55 (32.3%), 79 (46.5%), and 28 (16.5%), attained tertiary, secondary, and primary levels of formal education, respectively. On the other hand, 8 (4.7%) did not acquire formal education. Moderate LUTS (IPSS 8-19) was documented in 149 (87.6%) patients and severe LUTS (IPSS ≥20) in 21 (12.4%) patients. The mean age, BMI, tPSA, IPSS, prostate volume (PV), and Qmax are presented in Table 1. Additionally, 51 (30%) participants presented with a Qmax value of 15ml/s or less, 22 (12.9%) participants presented with PVR greater than 150ml, and 108 (63.5%) had median lobe protrusion. Regarding the quality of life due to the LUTS question, 92 (54.1%) participants responded “mostly satisfied” with their LUTS, and 63 (37.1%) reported “mixed” feelings regarding their LUTS. Only 9 (5.3%) participants were “mostly dissatisfied” and 6 (3.5%) participants were “unhappy” with their LUTS. No participant felt “terrible” about their LUTS. About 91.8% (156) of men were in marriages.

**Table 1:** baseline characteristics of participants are shown

Variable	Mean ± SD	Median (IQR)	Minimum	Maximum
Age (years)	63.70 ± 9.92	64 (57 - 70)	42	88
BMI (Kg/m^2^)	28.75 ± 3.61	28.7 (26.1 - 31.0)	18.64	40.00
IPSS	13.64 ± 4.14	13 (10 - 16)	8	23
tPSA (ng/ml)	2.68 ± 0.91	2.8 (2.1 - 3.5)	0.10	4.00
PV (cm^3^)	70.84 ± 39.50	66.3 (40.6 - 92.0)	16.00	273.00
Qmax (ml/s)	20.25 ± 9.70	18.0 (14.0 - 24.0)	5.00	50.00
PVR (ml)	77.66 ± 69.30	60.5 (25.0 - 100.8)	4.00	382.90

IPSS: International Prostate Symptom Score; tPSA: serum total prostate-specific antigen; PV: prostate volume; Qmax: peak urine flow rate; PVR: postvoid residual urine volume; SD: standard deviation; IQR: interquartile range; BMI: body mass index

**Correlation between Interviewer-assisted IPSS and (PVR and Qmax):** the positive correlation between PVR and IPSS (F: 32.91; r: 0.49; p <0.001), and the negative correlations between Qmax and IPSS (F: 31.27; r: -0.40; p <0.001) and between the level of formal education attained and IPSS (r: -0.23; p = 0.003) were respectively weak.

**Comparing means of PVR and IPSS using Qmax cut-off of 15ml/s:** the mean PVR of participants with Qmax >15ml/s (59.4±46.1ml) was significantly lower than the mean PVR of participants with Qmax ≤15ml/s (120.3 ± 92.6ml) (p= 0.001), indicating a larger PVR in patients with Qmax ≤15ml/s. Similarly, the mean IPSS of participants with Qmax >15ml/s (12.4±3.4) was significantly lower than the mean IPSS of participants with Qmax ≤15ml/s (16.4±4.4) (p 0.001), indicating a severe degree of IPSS in patients with Qmax ≤15ml/s.

**Determining the nature of the association between study variables and IPSS:** the associations of the other variables of interest (age of participant, highest level of formal education attained, marital status, body mass index (BMI), tPSA, Qmax, PV, PVR, median lobe protrusion, and QoL due to LUTS) and the reported IPSS were evaluated using multivariate logistic regression analysis (Table 2). The linear regression model generated was a significant predictor of the outcome variable [R2 0.665, F (10,158) =31.422, p<0.001].

**Table 2:** multivariate logistic regression analysis of study variables in relation to interviewer-assisted IPSS for lower urinary tract symptoms (LUTS)

Variables	Beta	T	95% CI of Beta	p-value
Age of participant (years)	-0.062	-1.126	-0.071 - 0.021	0.262
Highest level of formal education	-0.134	-2.728	-1.160 - -0.196	0.004
Marital status	0.031	0.616	-0.493 - 1.376	0.345
QoL due to LUTS	0.613	11.878	2.738 - 4.035	0.001
tPSA	0.050	1.057	-0.254 - 0.653	0.319
BMI	-0.011	-0.245	-0.118 - 0.090	0.801
Qmax	-0.153	-2.901	-0.114 - -0.022	0.009
PVR	0.206	3.939	0.006 - 0.020	0.005
Prostate volume	-0.030	-0.624	-0.014 - 0.007	0.524
Median lobe protrusion	-0.055	-1.082	-1.342 - 0.372	0.281

QoL: quality of life; LUTS: lower urinary tract symptoms; tPSA: serum total prostate-specific antigen; BMI: body mass index; Qmax: peak urine flow rate; PVR: postvoid residual urine volume

## Discussion

Though there are reports that men with BPE tend to present late with complications of the disease condition, some men can present for urologic care at the stage of LUTS only [2,14]. This study demonstrates that these men cut across various ages, formal education, and symptom severity strata. This is similar to the report by Kaplan and colleagues which demonstrates that men with LUTS due to BPE seek urologic care for the LUTS at different points in the symptom progression [14].

The majority of the participants in this study and many other similar studies sought medical care for LUTS in the moderate IPSS range [15,16]. However, some other studies such as Hamza *et al*. report that more men seek care at the late stages of severe IPSS [17]. The mean IPSS at presentation for the participants in this study and in the study by Lammer *et al*. [16] in the Netherlands were 13.64±4.14 points and 16.5±6.8 points respectively. On the other hand, the mean IPSS for the cohort studied by Hamza *et al*. [17] in Northern Nigeria was 18.24±6.93 points. In all, these reports support the assertion that men with LUTS from BPE do present at a wide range of IPSS.

**Figure 1:**
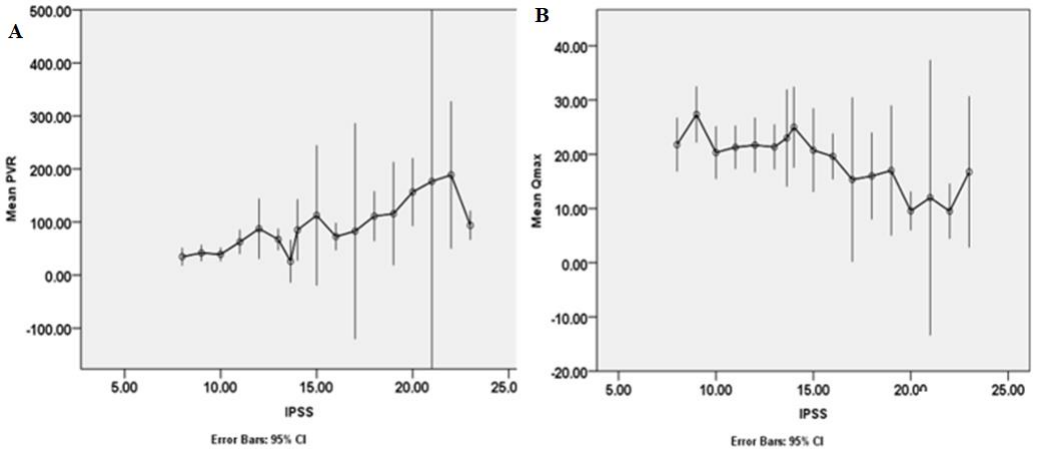
the plots of mean PVR against IPSS, and of mean Qmax against IPSS show; A) PVR was higher among the participants that check higher IPSS; B) Qmax was lower among these participants that check higher IPSS; Qmax: peak flow rate in ml/s; PVR: postvoid residual urine in ml; IPSS

From the pathophysiology of BOO secondary to BPE, worsening outlet obstruction is expected to result in decreasing Qmax values, and subsequent decompensation of the detrusor is expected to lead to increasing PVR values. Similarly, worsening lower urinary tract symptoms is expressed as increasing IPSS points and poorer QoL due to LUTS scores [18]. Figure 1 demonstrates that higher values of IPSS obtained through interviewer assistance are associated with progressively increasing PVR (figure 1A) as well as progressively decreasing Qmax (figure 1B). This pattern of relationship is reported in some other studies where there is no obvious evidence of interviewer assistance in determining IPSS [3,4,19]. On the other hand, some other studies report the absence of any clear pattern of relationship between self-administered IPSS and PVR and Qmax which are objective measures of BOO [5,16]. The lack of correlation between self-administered IPSS on the one hand and PVR and Qmax on the other hand in some studies may be due to poor comprehension of the language of the IPSS questionnaire by the responder.

In this study, lower IPSS points are significantly associated with Qmax greater than 15ml/sec (p = 0.001) implying that in the context of interviewer assistance, lower symptom scores are recorded in men with higher peak urine flow rates. Similarly, the PVR of men with Qmax greater than 15ml/sec is significantly lower (p = 0.001) than the PVR of participants with Qmax of 15ml/sec or less. These findings are observed despite the concerns of possible bias consequent upon interviewer assistance in determining IPSS. It is therefore appropriate to suggest that the observed discrepancies in the reported relationships between IPSS and PVR or IPSS and Qmax from different studies may be attributable to the subjectivity of IPSS, as an instrument for assessing the severity of LUTS from BOO rather than to the mode of administration of the IPSS questionnaire [20,21].

Put together, the highest level of formal education attained, and perceived quality of life due to LUTS, Qmax, and PVR are seen from this study to agree with the interviewer-assisted IPSS points (Table 2). The correlation is strong for the perceived QoL due to LUTS, but rather weak for Qmax and PVR. Despite the lower level of formal education for many of the participants and the absence of a validated IPSS questionnaire in the local language, there is significant agreement between the IPSS as administered with interviewer assistance and the reported QoL due to LUTS score.This finding is reassuring because the alternative strategy for deploying IPSS in low-formal education settings, in the form of a diagrammatic illustration of questionnaire items and options, known as visual prostate symptom score (VPSS) has not been found satisfactory [8,9]. Men who attained higher formal education appeared to seek urologic care for LUTS at lower symptom scores corroborating findings from an earlier community-based study that men who attained higher formal education tend to seek medical care for LUTS earlier [22]. Unit advancement in formal education predicts 13.4% of the variation in the lower urinary tract symptom score at presentation. Similarly, unit reduction in Qmax and unit increase in PVR predicts 15.3% and 20.6% respectively of the deterioration in IPSS at presentation. Expectedly, the highest contribution to the observed variation in IPSS amongst participants (about 61.3%) comes from the participants´ perceived QoL due to the LUTS score. There are no indications from this study that age at presentation, prostate volume as assessed using transrectal ultrasonography, body mass index (BMI), and serum total prostate-specific antigen (tPSA) can significantly predict interviewer-assisted IPSS. These findings do not differ from those from other studies on self-administered IPSS [15,23-26].

**Limitations:** this study is a single-center experience over a period of 12 months with the limitations inherent in such single-center questionnaire-based studies. In addition, men with IPSS <8 were excluded. There was no randomization as well. The findings therein can only be generalized with caution.

## Conclusion

Men who suffer LUTS from uncomplicated BPE present to urology care at varying degrees of LUTS severity. There is some evidence that IPSS administered with interviewer (physician) assistance correlates with Qmax and PVR urine volume in men with BOO due to BPE. The correlation is however weak. Perceived QoL due to LUTS makes the largest contribution to the variation in reported IPSS. In this low-resource and low-formal education setting, Qmax, PVR, QoL due to LUTS, and the level of formal education attained are observed to influence IPSS as determined with interviewer assistance. It is recommended that similar studies be conducted in other low-resource, low-formal education settings.

### What is known about this topic

- *Interviewer-administered IPSS has the potential to introduce bias due to the presence and role of the interviewer;*
- *In low formal education settings, self-administered IPSS is difficult to accomplish due to difficulty in understanding the items and their options;*
- *An alternative to self-administered IPSS, in the form of Visual Prostate Symptom Score (VPSS), has not eliminated the challenges with conventional self-administered IPSS in low-formal education settings*.

### What this study adds

- *Interviewer-assisted administration of IPSS in southeast Nigeria, a predominantly low-formal education, low-resource setting correlates with a peak flow rate in BOO due to BPE;*
- *Postvoid residual volume of urine (PVR) also correlates with IPSS determined via interviewer assistance;*
- *Evidence exits that interviewer-administered IPSS and PVR are significantly worse in men with Qmax of 15ml/sec or less when compared to men with Qmax greater than 15ml/sec*.

## References

[1] Madersbacher S, Sampson N, Culig Z (2019). Pathophysiology of Benign Prostatic Hyperplasia and Benign Prostatic Enlargement: A Mini-Review. Gerontology.

[2] Güler C, Tüzel E, Dogantekin E, Kiziltepe G (2008). Does sildenafil affect uroflowmetry values in men with lower urinary tract symptoms suggestive of benign prostatic enlargement?. Urol Int.

[3] Singla S, Garg R, Singla A, Sharma S, Singh J, Sethi P (2014). Experience with uroflowmetry in evaluation of lower urinary tract symptoms in patients with benign prostatic hyperplasia. J Clin Diagn Res.

[4] Wang JY, Liu M, Zhang YG, Zeng P, Ding Q, Huang J (2008). Relationship between lower urinary tract symptoms and objective measures of benign prostatic hyperplasia: a Chinese survey. Chin Med J (Engl).

[5] Mazzariol O, Reis LO, Palma PR (2019). Correlation of tools for objective evaluation of infravesical obstruction of men with lower urinary tract symptoms. Int Braz J Urol.

[6] Ugwumba FO, Ozoemena OF, Okoh AD, Echetabu KN, Mbadiwe OM (2014). Transvesical prostatectomy in the management of benign prostatic hyperplasia in a developing country. Niger J Clin Pract.

[7] McVary KT, Roehrborn CG, Avins AL, Barry MJ, Bruskewitz RC, Donnell RF (2011). Update on AUA guideline on the management of benign prostatic hyperplasia. J Urol.

[8] Schlatmann FWM, van Balken MR, de Winter AF, de Jong IJ, Jansen CJM (2022). How Do Patients Understand Questions about Lower Urinary Tract Symptoms? A Qualitative Study of Problems in Completing Urological Questionnaires. Int J Environ Res Public Health.

[9] Stothers L, Macnab A, Bajunirwe F, Mutabazi S, Lobatt C (2017). Comprehension and construct validity of the Visual Prostate Symptom Score (VPSS) by men with obstructive lower urinary tract symptoms in rural Africa. Can Urol Assoc J.

[10] Sarwar MR, Iftikhar S, Sarfraz M (2018). Influence of Education Level of Older Patients on Polypharmacy, Potentially Inappropriate Medications Listed in Beer's Criteria, and Unplanned Hospitalization: A Cross-Sectional Study in Lahore, Pakistan. Medicina (Kaunas).

[11] Barry MJ (2001). Evaluation of symptoms and quality of life in men with benign prostatic hyperplasia. Urology.

[12] Porru D, Bartoletti R, Austoni E, Carrino M, Gianneo E, Melloni D (2001). Relationship of flow rate with symptoms, quality of life and other clinical parameters in patients with LUTS suggestive of BPH. Eur Urol.

[13] Browner WS, Newman TB, Hulley SB (2007). Estimating Sample Size and Power: Application and Examples. Designing clinical research.

[14] Kaplan SA, Kohler TS, Kausik SJ (2020). Noninvasive Pressure Flow Studies in the Evaluation of Men with Lower Urinary Tract Symptoms Secondary to Benign Prostatic Hyperplasia: A Review of 50,000 Patients. J Urol.

[15] Güzel Ö, Aslan Y, Balci M, Tuncel A, Keten T, Erkan A (2015). Can Bladder Wall Thickness Measurement Be Used for Detecting Bladder Outlet Obstruction?. Urology.

[16] Lammers HA, Teunissen TAM, Bor H, Smid IS, Lagro-Janssen ALM (2020). No Relationship Between the International Prostate Symptom Score and Post-Void Residual Volume in Primary Care. Res Rep Urol.

[17] Hamza BK, Ahmed M, Bello A, Tolani MA, Awaisu M, Lawal AT (2021). Correlation of intravesical prostatic protrusion with severity of lower urinary symptoms among patients with benign prostatic hyperplasia. Afr J Urol.

[18] de Zeeuw S, Hop W, Huang Foen Chung J, van Mastrigt R (2014). Longitudinal changes in isovolumetric bladder pressure in response to age-related prostate growth in 1,020 healthy male volunteers. Neurourol Urodyn.

[19] Bosch JL, Hop WC, Kirkels WJ, Schröder FH (1995). The International Prostate Symptom Score in a community-based sample of men between 55 and 74 years of age: prevalence and correlation of symptoms with age, prostate volume, flow rate and residual urine volume. Br J Urol.

[20] Bozlu M, Doruk E, Akbay E, Ulusoy E, Cayan S, Acar D (2002). Effect of administration mode (patient vs physician) and patient's educational level on the Turkish version of the International Prostate Symptom Score. Int J Urol.

[21] Cam K, Akman Y, Cicekci B, Senel F, Erol A (2004). Mode of administration of international prostate symptom score in patients with lower urinary tract symptoms: physician vs self. Prostate Cancer Prostatic Dis.

[22] Nnabugwu II, Okoronkwo IL, Nnabugwu CA (2020). Lower urinary tract symptoms in men: challenges to early hospital presentation in a resource-poor health system. BMC Urol.

[23] Choi CK, Kim SA, Jeong JA, Kweon SS, Shin MH (2019). Non-linear Relationship Between Body Mass Index and Lower Urinary Tract Symptoms in Korean Males. J Prev Med Public Health.

[24] Telli O, Demirbas A, Kabar M, Karagoz MA, Sarici H, Resorlu B (2015). Does Metabolic Syndrome or its Components Correlate With Lower Urinary Tract Symptoms in Benign Prostatic Hyperplasia Patients?. Nephrourol Mon.

[25] Russo GI, Castelli T, Urzì D, Privitera S, Fragalà E, La Vignera S (2015). Connections between lower urinary tract symptoms related to benign prostatic enlargement and metabolic syndrome with its components: a systematic review and meta-analysis. Aging Male.

[26] Tsukamoto T, Masumori N, Rahman M, Crane MM (2007). Change in International Prostate Symptom Score, prostate-specific antigen and prostate volume in patients with benign prostatic hyperplasia followed longitudinally. Int J Urol.

